# Assessment of bioactive peptides derived from laminin-111 as prospective breast cancer-targeting agents

**DOI:** 10.1007/s00726-023-03379-x

**Published:** 2024-01-29

**Authors:** Fernanda Ferreira Mendonça, Danielle Vieira Sobral, Ana Claudia Ranucci Durante, Ana Cláudia Camargo Miranda, Jorge Mejia, Daniele de Paula Faria, Fabio Luiz Navarro Marques, Marycel Figols de Barboza, Leonardo Lima Fuscaldi, Luciana Malavolta

**Affiliations:** 1 Department of Physiological Sciences, Santa Casa de Sao Paulo School of Medical Sciences, Rua Dr. Cesareo Motta Jr. 61, Sao Paulo, CEP 01221-020 Brazil; 2 Hospital Israelita Albert Einstein, Sao Paulo, 05521-200 Brazil; 3 Laboratory of Nuclear Medicine (LIM-43), Department of Radiology and Oncology, Faculdade de Medicina FMUSP, Universidade de Sao Paulo, Sao Paulo, 01246-903 Brazil

**Keywords:** Laminin 111-derived peptides, Targeting molecules, Radiolabeled peptides, Breast cancer

## Abstract

Breast cancer remains a pressing public health issue primarily affecting women. Recent research has spotlighted bioactive peptides derived from laminin-111, implicated in breast tumor development. Remarkably, the sequences IKVAV, YIGSR, and KAFDITYVRLKF from the α1, β1, and γ1 chains, respectively, have garnered significant attention. This study aims to assess the potential of these radiolabeled peptides as targeting agents for breast cancer. The three peptides were synthesized using the Fmoc strategy, purified via reversed-phase high-performance liquid chromatography (RP-HPLC), and characterized through mass spectrometry. Iodine-131 (^131^I) radiolabeling was performed using the chloramine T method, exhibiting high radiochemical yield and stability for [^131^I]I-YIKVAV and [^131^I]I-YIGSR. Conversely, [^131^I]I-KAFDITYVRLKF demonstrated low radiochemical yield and stability and was excluded from the biological studies. The lipophilicity of the compounds ranged from − 2.12 to − 1.10. Serum protein binding assay for [^131^I]I-YIKVAV and [^131^I]I-YIGSR reached ≅ 48% and ≅ 25%, respectively. Affinity for breast cancer cells was evaluated using MDA-MB-231 and MCF-7 tumor cell lines, indicating the affinity of the radiopeptides with these tumor cells. Ex vivo biodistribution profiles of the radiopeptides were assessed in the MDA-MB-231 breast tumor animal model, revealing tumor tissue accumulation, supported by a high tumor-to-contralateral muscle ratio and autoradiography. These results signify the effective penetration of YIKVAV and YIGSR into tumor tissue. Therefore, the synthesized α1 and β1 peptide fragments exhibit favorable characteristics as potential breast cancer-targeting agents, promising future exploration as radiopharmaceuticals for breast cancer.

## Introduction

Cancer stands as a significant and urgent global public health concern. In 2020, the Global Cancer Observatory reported a staggering 9.2 million newly diagnosed cancer cases and 4.4 million cancer-related death among females. For women, breast cancer emerges as the predominant malignancy, accounting for around 2.3 million fresh instances (24.5%) and 682 thousand fatalities (15.5%). This prominence of breast cancer underscores a substantial challenge within the public health system, given its high mortality rate (Batiston et al. 2011; Ferlay et al. 2021).

The progression of tumorigenesis and subsequent tumor development involves an intricate interplay of molecular changes (Montor et al. 2018). In the context of breast cancer, uncontrolled cellular growth gives rise to proliferative potential, sustained angiogenesis, and metastatic competence (Hanahan and Weinberg 2011). Beyond the inherent modifications in tumor cells, the surrounding microenvironment significantly contributes to the carcinogenic process. A prime example is furnished by the laminin family of glycoproteins, which are a pivotal component of the extracellular matrix. While critical for tissue morphogenesis and homeostasis, laminins influence tumor invasion, angiogenesis, and metastasis (Ponce et al. 2001; Bosman and Stamenkovic 2003; Hamill et al. 2009).

Laminin-111, comprising α1, β1, and γ1 chains, is intricately linked to pivotal cellular processes, including adhesion, differentiation, proliferation, protease secretion, and metastasis within tumor cells. Additionally, bioactive peptides derived from laminin-111 have been identified as agents that influence the malignancy of tumors (Kikkawa et al. 2013; Otagiri et al. 2013).

Within the spectrum of biologically active peptides derived from laminin-111, a prominent role is assumed by the IKVAV peptide, sourced from the α1 chain. This peptide engenders cell adhesion and migration, thereby wielding a pivotal influence on tumor growth, metastasis, protease secretion, and angiogenesis across various tumor types (Sweeney et al. 1991; Bresalier et al. 1995; He et al. 2015). Extracted from the β1 chain, the CDPGYIGSR fragment emerges as a key entity associated with angiogenesis inhibition in diverse tumor contexts. Remarkably, subsequent investigations have illuminated that even the truncated YIGSR sequence retains its biological efficacy (Tashiro et al. 1989; Ponce et al. 2003; De Souza Santos 2011). Finally, the KAFDITYVRLKF sequence, known as the C16 peptide, represents the γ1 chain and is associated with an indelible imprint on gene expression in cells originating from invasive ductal carcinoma of the breast (MDA-MB-231) (Mokotooff et al. 1997; Smuczek et al. 2017).

As outlined in the literature, the laminin-111-derived peptides IKVAV, YIGSR, and KAFDITYVRLKF exhibit specific interactions with cell membrane receptors, namely integrins α_3_β_1_ and α_6_β_1_, 67 KD protein, and integrins α_v_β_3_ and α_5_β_1_, respectively (Kikkawa et al. 2013). Notably, the expression of these receptors is upregulated on the surface of tumor cells compared to their physiological presence on normal cell surfaces (Schottelius and Wester 2009). This divergence in expression levels suggests a potential avenue for employing these receptors as targets and the associated peptides as targeting agents for therapeutic or diagnostic interventions at tumor sites.

In this context, the present study aims at assessing the viability of peptides derived from the α1 (IKVAV), β1 (YIGSR), and γ1 (KAFDITYVRLKF) chains of laminin-111, radiolabeled with iodine-131 (^131^I), as prospective targeting agents for breast cancer.

## Materials and methods

### Peptides synthesis

The YIKVAV (α1 chain), YIGSR (β1 chain), and KAFDITYVRLKF (γ1 chain) peptides were synthesized manually by Solid Phase Peptide Synthesis (SPPS) method, using the Fmoc strategy (Fields and Noble 1990), at 1.0 mmol scale on Wang resin (0.70 mmol/g). The amino acids and Wang resin were purchased from Bachem (Torrance, USA). The α-amino group deprotection was carried out using a solution containing 2 M 4-methyl-piperidine in dimethylformamide (DMF), with stirring for 20 min, at room temperature. The coupling reaction was carried out using 2.5 excess of Fmoc-amino acid in the presence of 2.5 mM N,N′-diisopropylcarbodiimide (DIC)/2.5 mM hydroxybenzotriazole (HOBt) in a 1:1 (v/v) dichloromethane (DCM)/DMF mixture as a solvent system. Each coupling reaction was qualitatively verified using the ninhydrin test (Kaiser et al. 1970) after 2 h. At the end of the synthesis, a cleavage reaction was performed in a highly concentrated trifluoracetic acid (TFA) solution.

### Peptides characterization

All reagents and solvents meet the American Chemical Society standards or high-performance liquid chromatography (HPLC) grade.

#### Analytical and preparative RP-HPLC

The analyses and purification were conducted through RP-HPLC, using a C18 column (4.6 × 150 mm; particle size of 5 µm; pore size of 300 Å). Mobile phase A consisted of 0.1% TFA in H_2_O, while mobile phase B was 0.1% TFA in 60:40 acetonitrile (ACN)/H_2_O. The gradient profile for mobile phase B encompassed 5–95% in 30 min; flow rate of 1.0 mL/min for analytical purposes and 10.0 mL/min for purification purposes, with signal acquisition performed via a UV detector (λ = 220 nm).

#### Liquid chromatography mass spectrometry (LC–MS)

A system consisting of a micromass platform LCZ spectrometer, a Waters Alliance HPLC, a Waters 996 Photodiode Array detector and a Compaq workstation was used to characterize the synthesized peptides. A Waters Nova-Pak C18 column (2.1 × 150 mm; particle size of 3.5 μm; pore size of 60 Å) was employed, using the mobile phase A consisted of 0.1% TFA in H_2_O, while mobile phase B was 0.1% TFA in 60:40 ACN/H_2_O. The gradient profile for mobile phase B encompassed 5–95% in 30 min; flow rate of 0.4 mL/min. The detection was at 220 nm over a mass range of 500–3930 Da.

### Radiolabeling of peptides

**Fig. 1:**
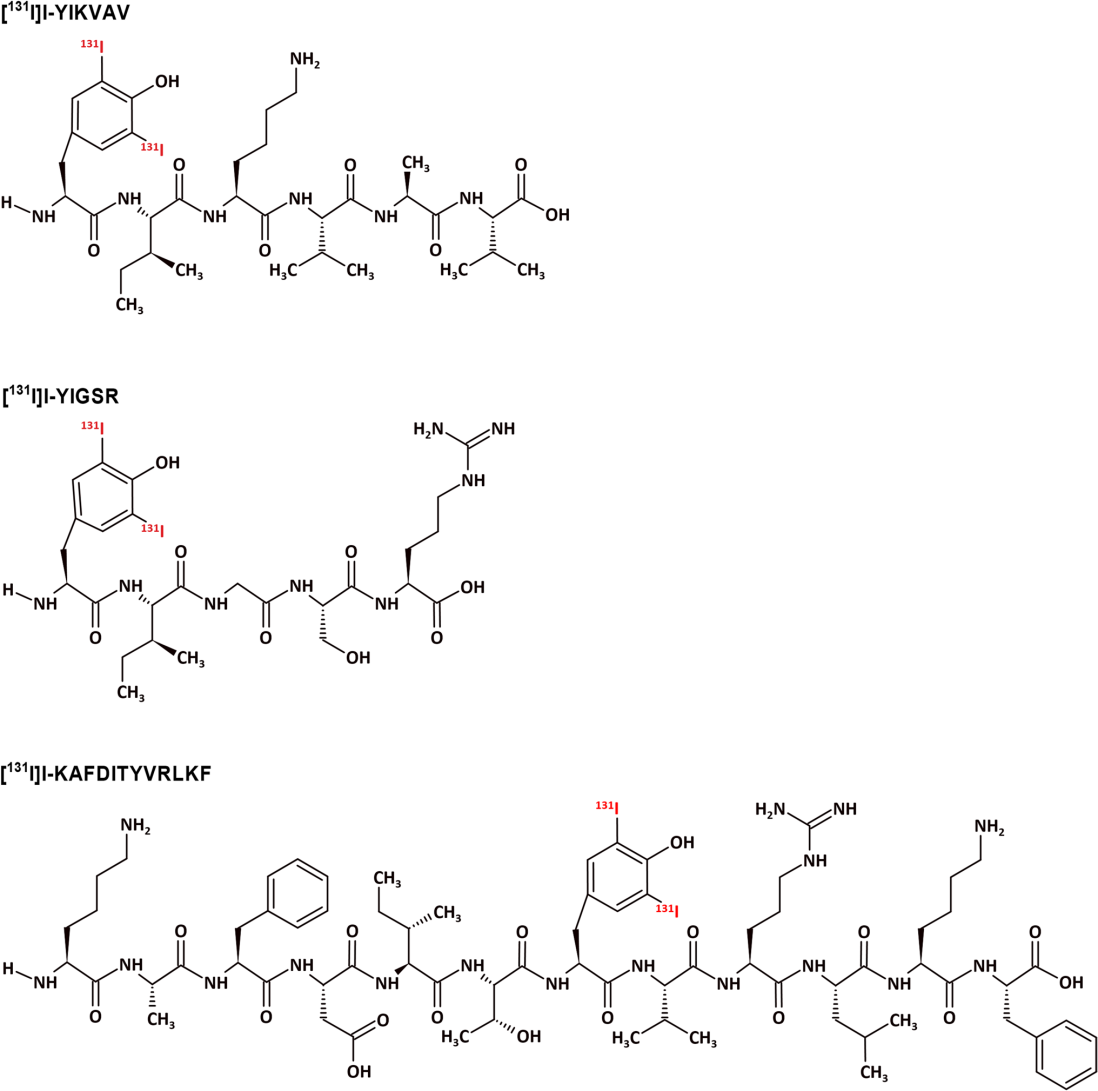
^131^I-labeled YIKVAV, YIGSR, and KAFDITYVRLKF peptides

The peptides were radiolabeled with ^131^I through the utilization of [^131^I]NaI and the chloramine T technique, as previously described (Durante et al. 2019). The ^131^I was obtained as [^131^I]NaI from Nordion (Ottawa, Canada) and distributed in Brazil by the *Instituto de Pesquisas Energéticas e Nucleares* of the *Comissão Nacional de Energia Nuclear*—*IPEN-CNEN* (Sao Paulo, Brazil). Briefly, the procedure involved introducing aliquots of approximately 11–15 MBq of [^131^I]NaI, with pH 7 for YIKVAV and 9–10 for YIGSR and KAFDITYVRLKF, and 3–5 μL of 1 mg/mL (4.4 mM) chloramine T solution into a vial containing a 25 μg aliquot (~ 36 nmol, 42 nmol and 17 nmol, respectively) of each peptide dissolved in a 0.1 M phosphate buffer solution (PBS) at pH 7.4. After 1 to 4 min, at room temperature, 5 µL of a solution containing 2 mg/mL (0.01 M) sodium metabisulfite was added to interrupt the radioiodination reaction. Figure 1 shows the chemical structure of the peptides after the ^131^I-labeling process.

The radiolabeling efficiency of the [^131^I]I-peptides was evaluated through an ascending chromatography technique employing silica gel-coated plates on an aluminum base (TLC-SG). Various mobile phases were assessed to determine the peptide radiolabeling yield. Following standardization, the solvent system that yielded superior separation between the [^131^I]I-peptides and [^131^I]NaI was constituted of 95:5 ACN/H_2_O for [^131^I]I-YIKVAV and [^131^I]I-YIGSR, whereas for [^131^I]I-KAFDITYVRLKF the better mobile phase was 0.9% NaCl. After the chromatographic development, the distribution of radioactivity on the strip was determined using a radiation scanner. The retention factors (R_f_) of [^131^I]NaI and [^131^I]I-peptides, along with the percentage of activity corresponding to the respective peaks, were ascertained.

The radiochemical yield was also assessed using RP-HPLC analyses. A C18 analytical column (3.0 × 100 mm; 2.6 µm) maintained at 30 °C by a column oven was employed. The mobile phase A consisted of 0.1% TFA in H_2_O, while mobile phase B comprised 0.1% TFA in ACN. The gradient profile for mobile phase B encompassed the following proportions: 3–10% (0–2 min), 10–30% (2–16 min), and 30–5% (16–22 min); flow rate of 1.0 mL/min, with signal acquisition being performed via both a UV detector (λ = 220 nm) and a radioactivity detector. In the chromatographic analysis, the retention times (R_t_) corresponding to unlabeled peptides, [^131^I]NaI, and [^131^I]I-peptides were determined.

### Radiochemical stability

The radiochemical stability of the radiopeptides was evaluated in two distinct environments: in saline (*n* = 3), at room temperature and under refrigeration (4–8 °C), by a period of 48 h after the radiolabeling, and in serum (*n* = 5), at 37 °C, for up to 24 h after incubation. At predefined time intervals, aliquots were extracted and subjected to ascending chromatography, as previously detailed.

### Lipophilicity

The lipophilicity was evaluated according to established methods (Durante et al. 2019). Briefly, 25 µL of each radiopeptide (0.6 MBq) were added into a blend of *n*-octanol (500 µL) and water (475 µL), and vortexing for 30 s, followed by centrifugation (5000 rpm) for 5 min (*n* = 5). From both phases, 100 µL aliquots were withdrawn and measured employing an automatic gamma counter. The lipophilicity was quantified as Log P value [Eq. (1)]:

1 $$P = log \, \left[\frac{cpm \, {\text{(organic phase}})}{cpm \, {\text{(aqueous phase}})}\right]$$

### Serum protein binding (SPB)

The percentage of radiopeptides bound to serum proteins was quantified according to the literature (Sobral et al. 2020). Briefly, 25 μL of each radiopeptide (0.6 MBq) were added into 475 μL of serum and incubated (37 °C) under gentle agitation for 1 h (*n* = 5). Subsequently, 1 mL of 0.6 M trichloroacetic acid (TCA) was added, vortexing for 1 min, and the samples were centrifuged (4000 rpm) for 10 min. The supernatant and the pellet were separated, and the radioactivity in each fraction was measured in an automatic gamma counter. The SPB percentage was calculated as [Eq. (2)]:

2 $$SPB \,[\%]=\frac{cpm (pellet)}{cpm (pellet+supernatant)}\times 100$$

## Biological evaluation

### Culture of breast cancer cells (MDA-MB-231 and MCF-7)

The human breast cancer cell lines were purchased from the *Banco de Células do Rio de Janeiro*—*BCRJ* (Rio de Janeiro, Brazil). The MDA-MB-231 and MCF-7 cells were cultured in RPMI-1640 and DMEM-F12, respectively, supplemented with 10% (v/v) fetal bovine serum and 1% (v/v) antibiotics (penicillin and streptomycin). The cells were incubated in a 5% CO_2_ atmosphere, at 37 °C, and grown to a 90% confluence. Then, cells were trypsinized, centrifuged (1,500 rpm) for 5 min, and resuspended in their respective supplemented medium for the in vitro assays. For the development of the breast cancer animal model, MDA-MB-231 cells were resuspended in 1:1 matrigel:RPMI-1640 mixture.

### Binding and internalization studies of the radiopeptides to MDA-MB-231 and MCF-7 tumor cells

In vials containing 2 × 10^6^ MDA-MB-231 or MCF-7 cells resuspended in supplemented medium (450 μL), 50 μL of the [^131^I]I-peptides (1.2 MBq) were added and incubated at 37 °C under gentle agitation (500 rpm) for 1, 4 and 24 h (*n* = 5 for each time interval). Subsequently, the vials were centrifuged (5000 rpm) for 5 min and the supernatant was collected. The radioactivity of the cell pellet and supernatant was measured in an automatic gamma counter. The binding percentage was calculated as Eq. (3):

3 $$Binding \,[\%]=\frac{cpm \,(pellet)}{cpm \,(pellet+supernatant)}\times 100$$

After that, the pellets were resuspended in 0.5 mL of acid buffer (0.2 mM acetic acid in 0.5 M NaCl solution; pH 2.8) and kept at room temperature for 5 min to remove radiopeptides bound to the cell membrane surface. Then, the vials were centrifuged (5000 rpm) for 5 min and the supernatant was collected. The radioactivity of the cell pellet and supernatant was measured in an automatic gamma counter. The internalization percentage was calculated as Eq. (4):

4 $$Internalization \,[\%]=\frac{cpm \,(pellet)}{cpm \,(pellet+supernatant)}\times 100$$

### MDA-MB-231 breast tumor animal model

Female Balb/c nude mice (≅ 20 g; ≅ 8 w) were supplied and maintained in the vivarium of the *Centro de Experimentação e Treinamento em Cirurgia* of the *Hospital Israelita Albert Einstein* (Sao Paulo, Brazil), accredited by the Association for Assessment and Accreditation of Laboratory Animal Care (AAALAC) International. All procedures involving mice were approved by the Ethics Committee on Animal Use of the *Hospital Israelita Albert Einstein* (protocol #3740/19).

Mice were subcutaneously inoculated into the right lower flank with 200 µL of a solution containing 1 × 10^7^ MDA-MD-231 cells in a 1:1 matrigel:RPMI-1640 medium mixture. Tumors were allowed to grow in vivo for 30 days after inoculation or until reaching a diameter of 10 mm.

### Ex vivo* biodistribution profile and autoradiography*

The ex vivo biodistribution profiles of [^131^I]I-YIKVAV and [^131^I]I-YIGSR were determined after intravenous injection into MDA-MB-231 breast tumor-bearing (*n* = 4) and naive (*n* = 3) female Balb/c nude mice. Animals were anesthetized using ketamine–xylazine combination (100:10 mg/Kg) and euthanized at 15 and 120 min post-injection. Organs and tissues of interest were dissected, dried on filter paper, weighed, and measured using an automatic gamma counter. A standard dose with the same amount of radioactivity as injected into the mice was counted. The results were expressed as the percentage of injected dose per gram of tissue (%ID/g). After samples counting, the tumor and contralateral muscle were used for autoradiography as previously described (de Paula Faria et al. 2023). Briefly, tissues were frozen and sliced at 30 µm in a cryostat, then were exposed to a phosphor-imaging plate (BASMS-2325; Fujifilm, Japan) for 24 hats 21–25 °C. The plate was scanned using a Typhoon FLA 9500 plate reader (GE Healthcare, USA) with a pixel resolution of 100 µm for qualitative analysis.

### Statistical analysis

Quantitative data were expressed as either “mean ± standard deviation (SD)” (in vitro data) or “mean ± standard error of the mean (SEM)” (in vivo data). Pairs of groups were compared using the Student t-test. The means of three or more groups were compared by one-way Analysis of Variance (ANOVA), followed by Tukey’s post hoc test. The statistical significance threshold for mean differences was set at 0.05.

## Results

### Peptides synthesis

The synthesized YIKVAV (α1 chain), YIGSR (β1 chain), and KAFDITYVRLKF (γ1 chain) were obtained in approximately 70% yield. The theoretical molecular weights of 693.0 g/mol (YIKVAV), 596.0 g/mol (YIGSR), and 1501.0 g/mol (KAFDITYVRLKF) were confirmed by mass spectrometry.

### Radiolabeling of peptides and radiochemical stability

**Fig. 2:**
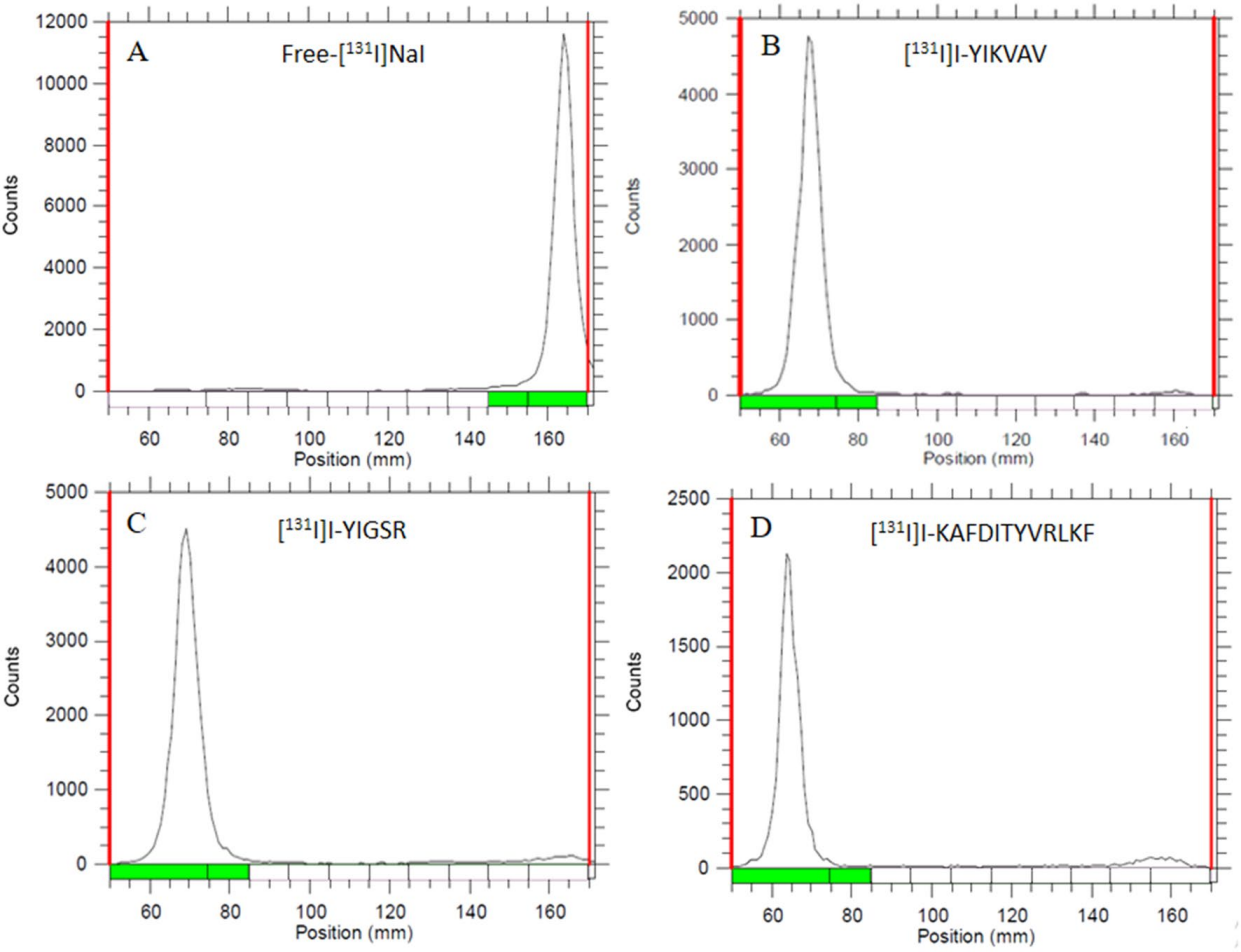
Ascending radiochromatograms obtained in TLC strips using either 95:5 ACN/H_2_O solution for free [^131^I]NaI, [^131^I]I-YIKVAV, and [^131^I]I-YIGSR, or 0.9% NaCl solution for [^131^I]I-KAFDITYVRLKF as eluents

**Fig. 3:**
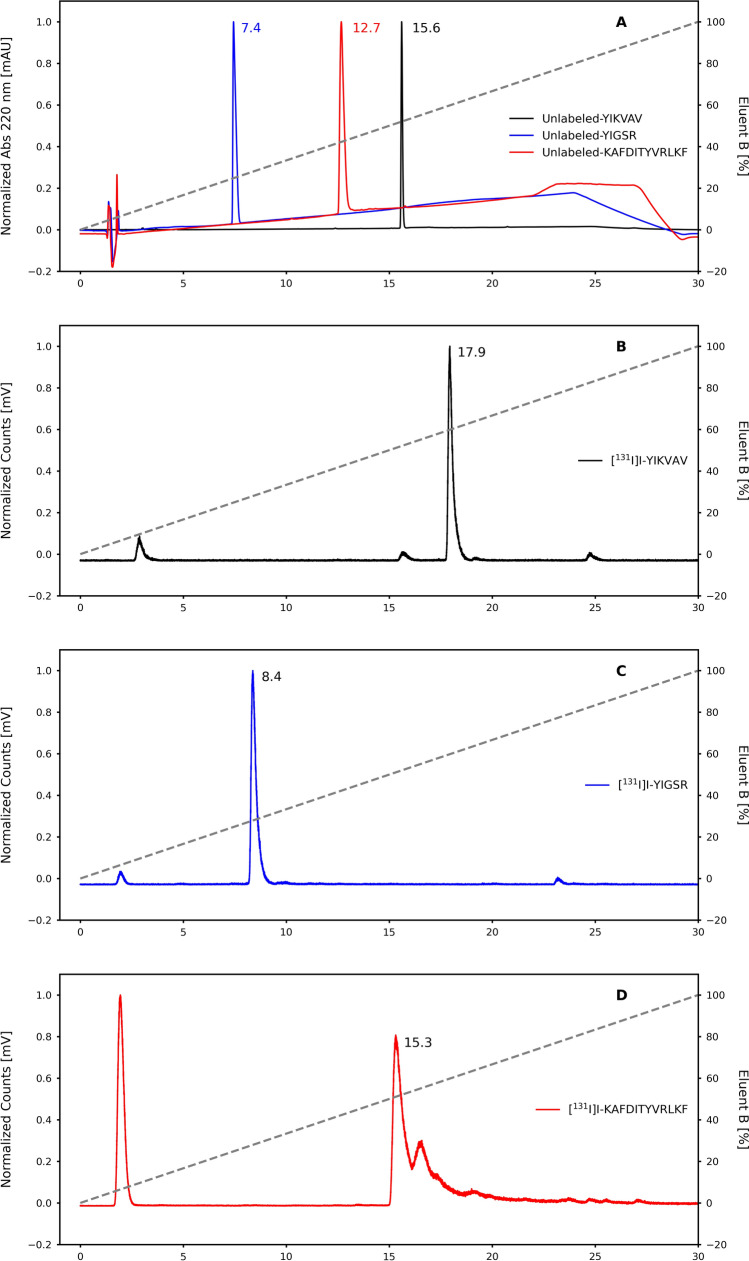
RP-HPLC profiles: **A** unlabeled YIKVAV (black line), YIGSR (blue line), and KAFDITYVRLKF (red line), **B** [^131^I]I-YIKVAV (black line), **C** [^131^I]I-YIGSR (blue line), and **D** [^131^I]I-KAFDITYVRLKF (red line)

The three peptides were ^131^I-labeled with radiochemical yields of 98 ± 4%, 93 ± 6%, and 80 ± 7% for [^131^I]I-YIKVAV (0.36 MBq/nmol), [^131^I]I-YIGSR (0.31 MBq/nmol), and [^131^I]I-KAFDITYVRLKF (0.76 MBq/nmol), respectively (*n* = 10), determined by TLC-SG chromatography (Fig. 2). In both chromatographic systems employed in this work, free [^131^I]NaI migrates with the mobile phase to the top of the strip (R_f_ = 0.9–1.0) and the radiolabeled peptide fragments remain at the origin of the strip (R_f_ = 0.1–0.2), allowing an efficient separation between [^131^I]I^−^ and radiolabeled species. The RP-HPLC analysis (Fig. 3A) showed R_t_ of 15.60, 7.44, and 12.67 min for unlabeled YIKVAV, YIGSR, and KAFDITYVRLKF, respectively, while the R_t_ of the correspondent labeled compounds were 17.94 (Fig. 3B), 8.37 (Fig. 3C), and 15.32 min (Fig. 3D), respectively. Free [^131^I]NaI showed R_t_ of 2.80 min.

**Fig. 4:**
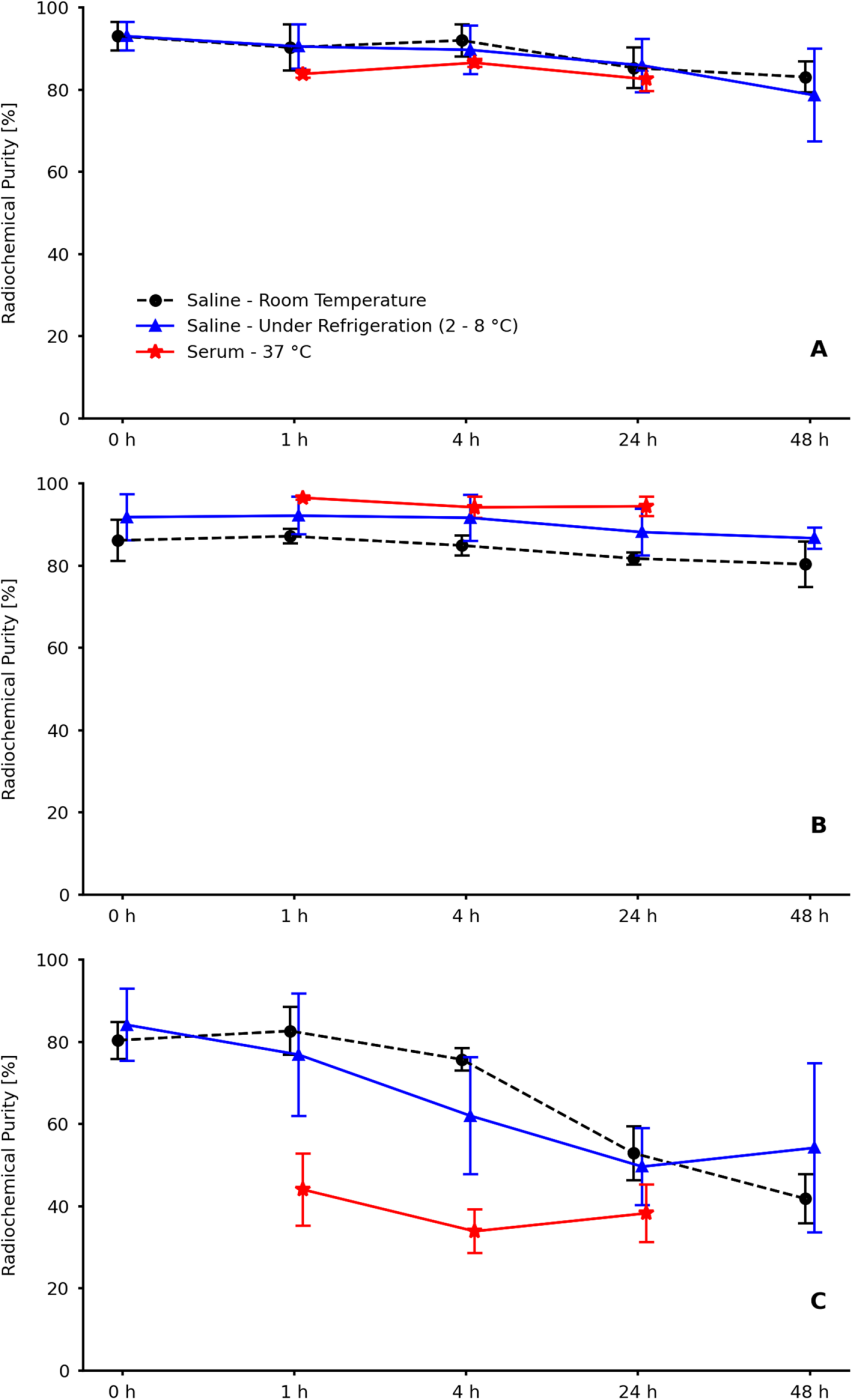
Radiochemical stability in saline and serum of **A**, **B** [^131^I]I-YIKVAV, **C**, **D** [^131^I]I-YIGSR, and **E**, **F** [^131^I]I-KAFDITYVRLKF. Data are expressed as ‘mean ± SD’ (*n* = 3 [saline]; *n* = 5 [serum]). Different letters represent statistically significant differences within time (p < 0.05)

The radiochemical stability of [^131^I]I-YIKVAV, [^131^I]I-YIGSR, and [^131^I]I-KAFDITYVRLKF are summarized in Fig. 4. The [^131^I]I-YIKVAV (Fig. 4A) and [^131^I]I-YIGSR (Fig. 4B) presented suitable radiochemical stability in saline and, only at 48 h post-radiolabeling, small reductions of ≅ 10 and 5%, respectively, were observed in the radiochemical purity. On the other hand, the radiochemical purity of [^131^I]I-KAFDITYVRLKF (Fig. 4C) decreased by approximately 50% after 24 h, when maintained at room temperature or under refrigeration. The radiochemical stability was also evaluated in serum, and the results were similar to those in saline, i.e., [^131^I]I-YIKVAV (Fig. 4A) and [^131^I]I-YIGSR (Fig. 4B) were stable within 24 h, but [^131^I]I-KAFDITYVRLKF (Fig. 4C) was unstable.

### Lipophilicity and serum protein binding (SPB)

The lipophilicity, expressed as Log P, and the SPB percentage data are summarized in Table 1.

**Table 1:** Lipophilicity (Log P) and serum protein binding (SPB) of the three ^131^I-labeled peptide fragments

Parameters	[^131^I]I-YIKVAV	[^131^I]I-YIGSR	[^131^I]I-KAFDITYVRLKF
Log P	− 1.61 ± 0.07	− 2.12 ± 0.08	− 1.10 ± 0.04
SPB [%]	48.40 ± 0.78	25.01 ± 0.70	nd

Data are expressed as ‘mean ± SD’ (*n* = 5)

*nd* not determined

### Biological evaluation

**Fig. 5:**
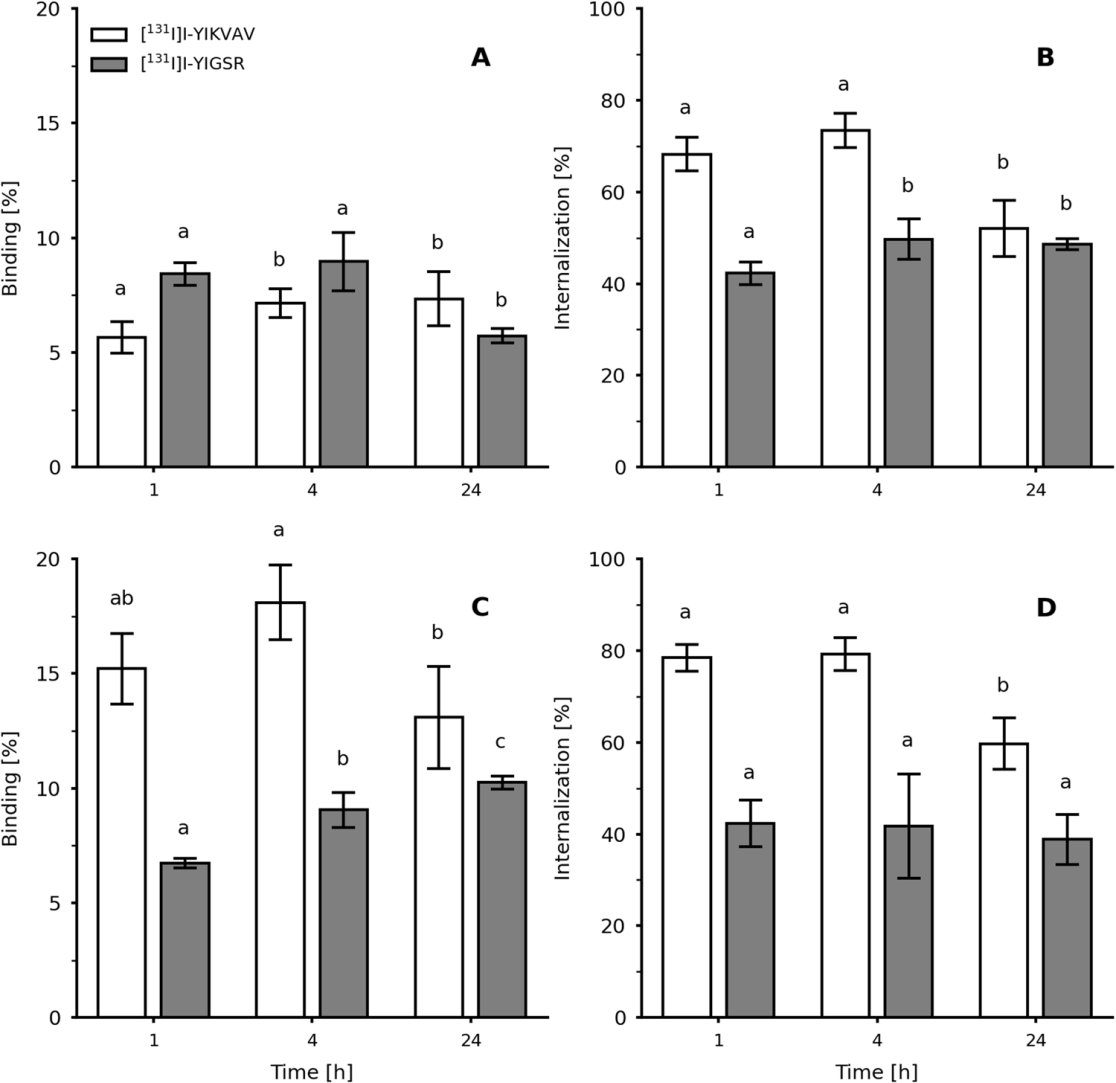
Binding and internalization percentages of the [^131^I]I-YIKVAV and [^131^I]I-YIGSR to **A**, **B** MDA-MB-231 and **C**, **D** MCF-7 breast cancer cells. Data are expressed as ‘mean ± SD’ (*n* = 5). Different letters represent statistically significant differences within time for the same radiopeptide (p < 0.05)

The affinity studies of [^131^I]I-YIKVAV and [^131^I]I-YIGSR were conducted in MDA-MB-231 and MCF-7 breast tumor cells **(**Fig. 5). The radiopeptides showed different values for the binding (Fig. 5A) and internalization (Fig. 5B) percentages to MDA-MB-231 cells. The binding percentages of [^131^I]I-YIKVAV were between 5.67 ± 0.68 and 7.34 ± 1.17% over the evaluated time interval. The [^131^I]I-YIGSR showed a binding percentage of 8.42 ± 0.50% at 1 h, which decreased to 5.72 ± 0.31% at 24 h. The internalization percentages of [^131^I]I-YIKVAV and [^131^I]I-YIGSR were 73.37 ± 3.73 and 49.70 ± 4.40%, respectively, at 1 h of incubation. Within 24 h, the internalization percentages of both [^131^I]I-YIKVAV and [^131^I]I-YIGSR were ≅ 52%. The radiopeptides also showed different results for the binding (Fig. 5C) and internalization (Fig. 5D) percentages to MCF-7 cells. The binding percentages of [^131^I]I-YIKVAV to MCF-7 cell line were approximately 15% throughout the incubation period, with a higher binding percentage of 18.10 ± 1.63% at 4 h. The [^131^I]I-YIGSR showed binding percentages from 6.67 ± 0.25% at 1 h to 10.02 ± 0.61% at 24 h. The internalization percentages of the [^131^I]I-YIKVAV were approximately 80% within the first hours of incubation and decreased to 59.71 ± 5.57% at 24 h. However, the [^131^I]I-YIGSR presented an internalization of approximately 40% within time.

**Fig. 6:**
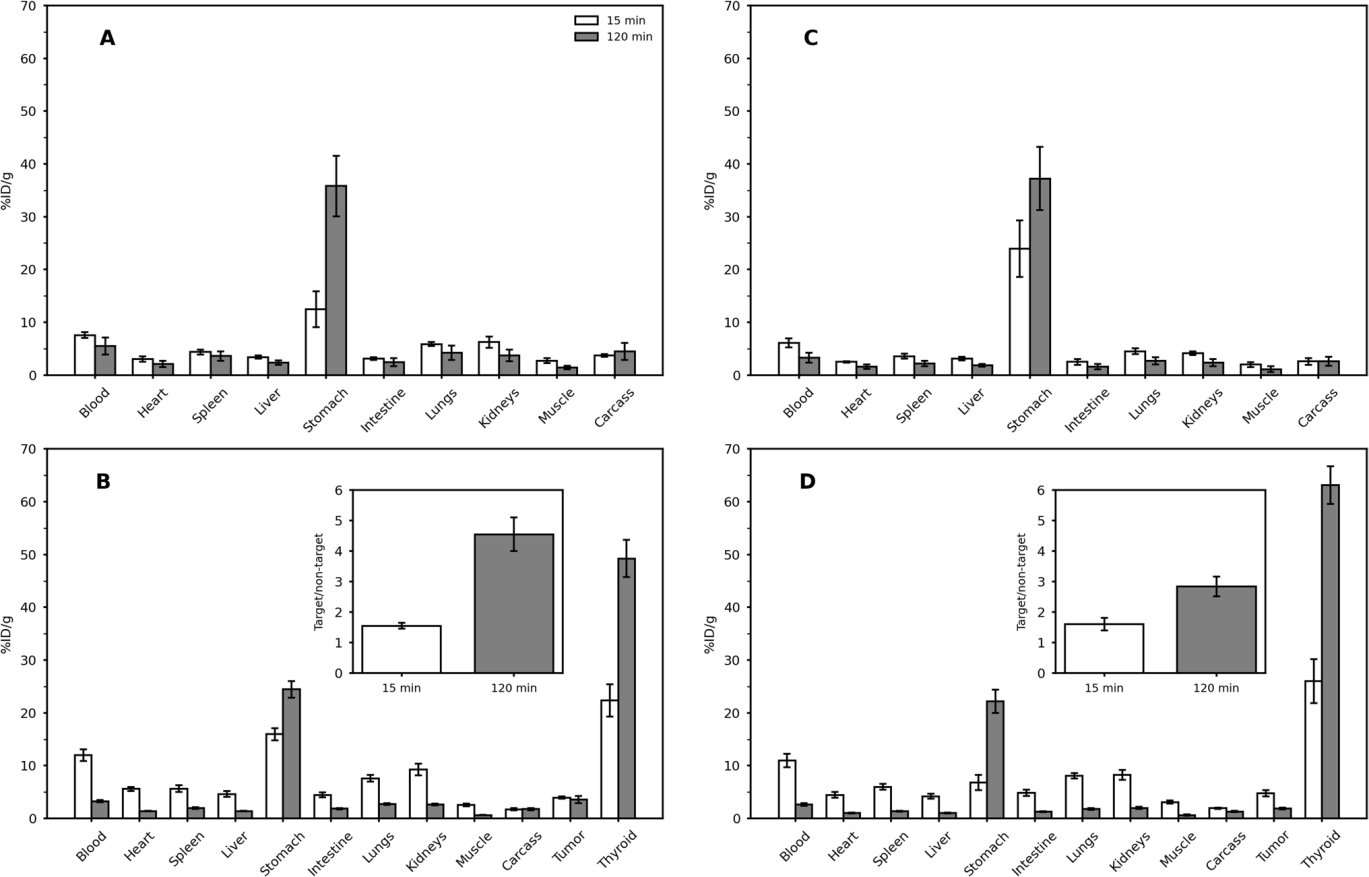
Ex vivo biodistribution of **A**, **B** [^131^I]I-YIKVAV and **C**, **D** [^131^I]I-YIGSR in naive (control) (*n* = 3) and MDA-MB-231 breast tumor-bearing nude female Balb/c mice (*n* = 5–7). Inserts: Tumor-to-contralateral muscle ratios obtained from the ex vivo biodistribution data in MDA-MB-231 breast tumor-bearing nude female Balb/c mice. Data are expressed as ‘mean ± SEM’

**Fig. 7:**
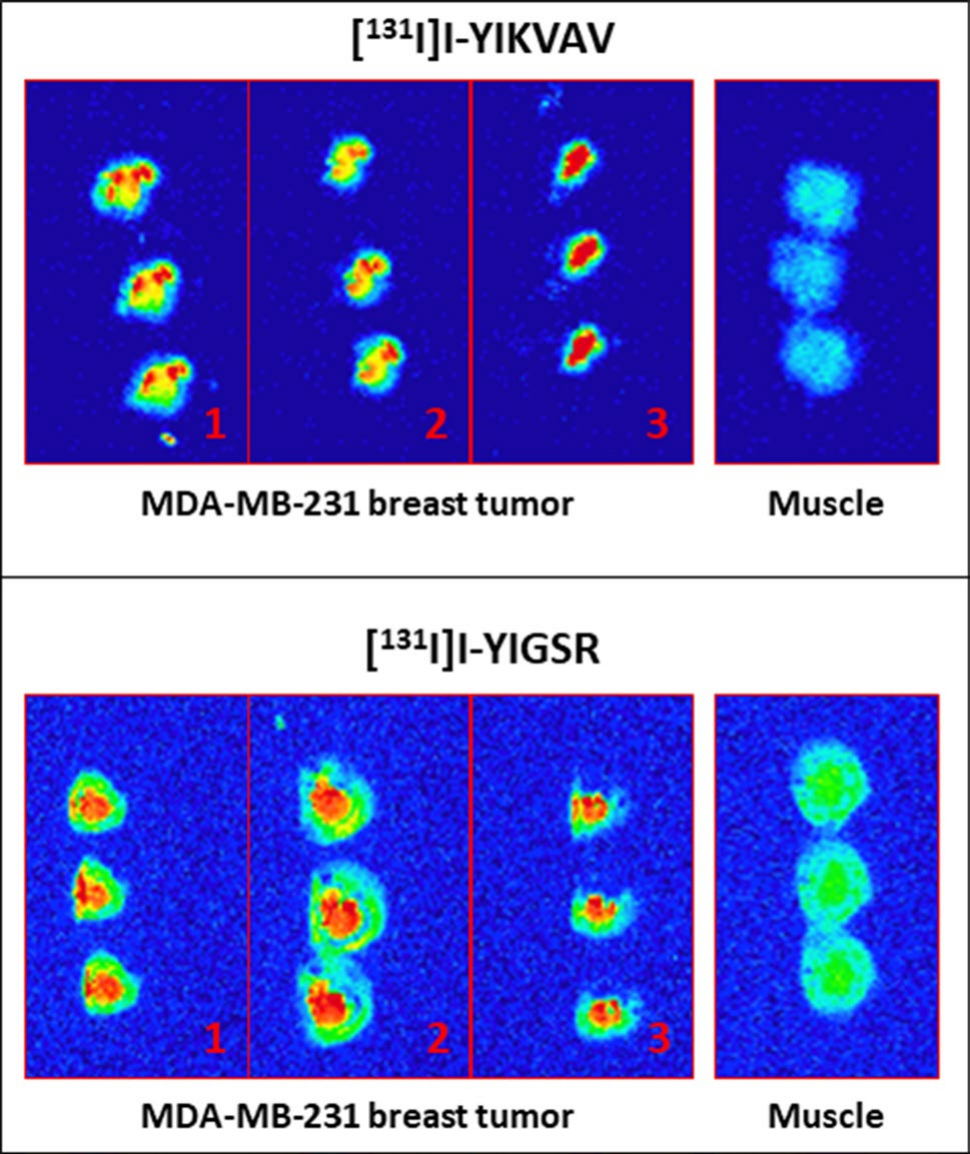
Autoradiography of [^131^I]I-YIKVAV and [^131^I]I-YIGSR in MDA-MB-231 breast tumor-bearing mice: (1) tumor center; (2) interior of the tumor between the center and the surface; (3) tumor surface

The [^131^I]I-YIKVAV and [^131^I]I-YIGSR ex vivo biodistribution profiles are summarized in Fig. 6. Both peptides exhibited rapid blood clearance and low accumulation in non-target organs. However, there was a notable accumulation of radioactivity in the stomach and thyroid. Data obtained from xenografted mice demonstrated that [^131^I]I-YIKVAV and [^131^I]I-YIGSR displayed high tumor accumulation. The tumor-to-contralateral muscle ratios (Fig. 6—inserts) were 1.5 at 15 min for both peptides, which increased to 4.6 and 2.8 at 120 min for [^131^I]I-YIKVAV and [^131^I]I-YIGSR, respectively. These findings were further supported by autoradiography (Fig. 7), where visual analysis of the images indicates a higher accumulation of radiopeptides in the tumor tissue than the contralateral muscle. Additionally, they were found to penetrate deeply into the tumor tissue.

## Discussion

Three laminin-111-derived peptide fragments were synthesized, each representing a different chain. The IKVAV fragment represented the α1 chain, modified by introducing the non-native amino acid tyrosine (Y) at the N-terminal position to allow the radiolabeling with ^131^I. Thus, the final structure is YIKVAV. The β1 chain was represented by the YIGSR fragment, a truncated version of the peptide CDPGYIGSR, which retained its biological activity, and the γ1 chain was represented by the C16 peptide, KAFDITYVRLKF. All fragments were synthesized using the Fmoc strategy and were purified by RP-HPLC, with chemical purity higher than 95%.

Radiolabeling of the peptides with ^131^I was accomplished by the chloramine T method (Hunter 1970; Ebenhan et al. 2018), with the radionuclide incorporated at the aromatic ring of the tyrosine aminoacid (Fig. 1). The choice of ^131^I in this study was influenced by its availability and cost-effectiveness.

In the radiolabeling procedure, the best radiochemical yields were achieved at 1.5, 1, and 4 min for YIKVAV, YIGSR, and KAFDITYVRLKF fragments, respectively. However, the γ1 representative, KAFDITYVRLKF, displayed a longer reaction time, likely due to steric hindrance caused by the central tyrosine residue (Fig. 1). Hu and co-workers have assessed the validity of labeling YIGSR with ^99m^Tc by employing the bifunctional chelator S-Acetyl-NH_3_-MAG3. This study achieved a radiolabeling yield of 62% (Hu et al. 2007). Consequently, our findings demonstrated that iodine radiolabeling offers a simpler procedure and higher radiochemical efficiencies.

The use of the ascending chromatography method to evaluate radiochemical yields was motivated by its ease of use, material availability and low cost associated with the procedure, and RP-HPLC analyses validated it. Due to the delay for the samples to go through from the UV detector to the radioactivity detector, the radiopeptides exhibited slightly extended R_t_ when compared to the unlabeled peptide fragments. Despite this, the RP-HPLC results align with the ascending chromatographic outcomes, providing further support for the accuracy and consistency of the obtained results.

The radiochemical stability of ^131^I-labeled peptides in saline was assessed at room temperature and under refrigeration (4–8 °C). In both conditions, the [^131^I]I-YIKVAV and [^131^I]I-YIGSR remained stable over time. However, the [^131^I]I-KAFDITYVRLKF decreased radiochemical purity to around 50%, within the specified time interval, for both storage conditions. The evaluation of radiochemical stability was extended to serum at 37 °C, for 24 h. Throughout the entire duration, the [^131^I]I-YIKVAV and [^131^I]I-YIGSR demonstrated consistent radiochemical stability within the serum. Conversely, the [^131^I]I-KAFDITYVRLKF exhibited radiochemical instability within the first hour of exposure to serum. The radiochemical stability is important when assessing a new peptide-based targeting molecule. Previous studies have illustrated that [^131^I]I-peptides, across various concentrations, maintained radiochemical stability for up to 24 h following the radiolabeling procedure (Araújo et al. 2009; Brunton et al. 2010).

All the radiopeptides displayed hydrophilic characteristics, supporting preferential renal excretion, which could generate a lower background for an imaging diagnosis radiopharmaceutical. Conversely, lipophilic compounds tend to be reabsorbed and recirculated within the systemic circulation (Jeghers et al. 1990). However, due to the lower radiochemical stability of the [^131^I]I-KAFDITYVRLKF, further evaluations were limited to the ^131^I-labeled YIKVAV and YIGSR. This prudent decision was guided by the necessity for stable radiolabeling, a crucial factor in the evaluation and utility of radiopeptides.

The findings from the SPB analysis revealed that ≅ 50% of the [^131^I]I-YIKVAV fragment and ≅ 75% of the [^131^I]I-YIGSR are available to reach the intended target. This indicates a considerable potential for clinical application, as these fractions signify the proportion of the peptides that could potentially engage in the desired interactions or therapeutic effects (de Barros et al. 2013).

The observed variations in the binding and internalization patterns of the radiopeptides within MDA-MB-231 and MCF-7 tumor cells likely stem from the unique characteristics of each evaluated peptide. The IKVAV peptide, recognized for its role in promoting cell adhesion and migration (Bresalier et al. 1995; He et al. 2015), is likely responsible for its higher binding and internalized fractions. Due to the importance of this fragment, a recent assessment was conducted on a lyophilized matrix composed of hyaluronic acid (HA) and the IKVAV peptide within a 3D in vitro model of breast cancer. The results demonstrated that the HA-IKVAV displayed elevated sensitivity compared to its 2D counterparts, particularly regarding cellular membrane permeabilization and viability, thereby reinforcing the significance of this fragment (Sieni et al. 2020). These attributes align with its tendency to facilitate interactions that promote these cellular behaviors.

On the contrary, the YIGSR peptide is associated with angiogenesis inhibition (Ponce et al. 2003). This aligns with our findings, as this peptide demonstrated the lowest percentage of both bound and subsequently internalized fractions within a 24 h incubation period. The effectiveness of this fragment in inhibiting the growth of melanoma and lung tumor cells has been under evaluation, including metastasis formation. These studies suggested that the YIGSR peptide have a potential to be used as targeting molecule for systemic delivery for the treatment of metastatic cancer (Iwamoto et al. 1988; Sarfati et al. 2011). Moreover, this pentapeptide-rhodamine B derivative (YIGSR-RhB) exhibited high absorption by B16F10 melanoma cells and 4T1 breast cancer cells, resulting in a robust fluorescent signal within these in vitro tumor cells and in vivo mice tumors. As a result, YIGSR-RhB exhibits promising potential as a tumor-targeting probe for fluorescent imaging, displaying the ability to directly adhere to the cell membrane and selectively target tumor cells (Liu et al. 2020). In the context of our results, it is evident that the proposed peptides exhibit favorable interactions in both types of breast cancer cell lines, thereby holding promise as potential breast cancer-targeting agents.

The in vivo assay conducted in naïve and breast tumor-bearing mice demonstrated that both [^131^I]I-YIKVAV and [^131^I]I-YIGSR exhibited rapid blood clearance and displayed low accumulation in various non-target organs. This desirable profile indicates a minimized radioactivity background. Nevertheless, notable radioactivity accumulation was observed in the stomach and thyroid, which can be attributed to the partial in vivo release of ^131^I from the peptides, generating free ^131^I as a radiochemical impurity that accumulates in those organs (Lisco et al. 2023). Conversely, the peptide fraction that retained radiolabeling exhibited substantial accumulation at the tumor site. This accumulation displayed a high targeted-to-non-targeted ratio that increased from 15 to 120 min, implying a remarkable specificity for tumor tissue. Previous studies reported targeted-to-non-targeted ratios for ^99m^Tc-labeled peptides in the same order of magnitude in a murine MCF-7 breast cancer model, however maximum tumor-to-muscle ratio was seen at 15 min post-injection. (Ahmadpour et al. 2018a, b). This specificity was further supported by autoradiography visual analysis, which illustrated higher peptide accumulation in the tumor tissue compared to the contralateral muscle at 120 min after peptide administration. Moreover, samples were obtained from various levels, ranging from the tumor surface to the center to assess the depth of penetration within the tumor tissue. These findings suggest that both YIKVAV and YIGSR peptides effectively reach and penetrate deep into the tumor tissue.

Given their promising attributes, both [^131^I]I-YIKVAV and [^131^I]I-YIGSR peptides are candidates for radiolabeling with more appropriate radioisotopes. This step could enhance their performance and facilitate their evaluation as potential radiopharmaceuticals. This avenue of exploration holds significant promise for advancing their clinical utility within the realm of breast cancer imaging diagnostic and treatment, which could be achieved by further chemical modifications to enable radiolabeling with other radionuclides.

## Conclusion

The YIKVAV, YIGSR and KAFDITYVRLKF peptide fragments were synthesized and radiolabeled with ^131^I. However, due to its limited radiochemical stability, the [^131^I]I-KAFDITYVRLKF fragment did not undergo further assessment. Conversely, the [^131^I]I-YIKVAV and [^131^I]I-YIGSR fragments displayed favorable radiochemical characteristics. The comprehensive in vitro and in vivo data indicated their potential as effective targeting agents for breast cancer.

## Data Availability

The authors confirm that the data supporting the findings of this study are available within the article. Raw data that support the findings of this study are available from the corresponding authors, upon reasonable request.
